# Assessment of Potential Toxic Effects of Fungicide Fludioxonil on Human Cells and Aquatic Microorganisms

**DOI:** 10.3390/toxics13050358

**Published:** 2025-04-30

**Authors:** Maria Antonopoulou, Anna Tzamaria, Sotiris Papas, Ioanna Efthimiou, Dimitris Vlastos

**Affiliations:** 1 Department of Sustainable Agriculture, University of Patras, GR-30131 Agrinio, Greece; annatz408@gmail.com; 2 Section of Genetics, Cell Biology and Development, Department of Biology, University of Patras, GR-26504 Patras, Greece; sotirispapas@outlook.com (S.P.); iefthimiou@upatras.gr (I.E.); dvlastos@upatras.gr (D.V.)

**Keywords:** fludioxonil, ecotoxicity, microalgae, bacteria, genotoxicity, cytotoxicity

## Abstract

Fludioxonil is a widely used fungicide that is frequently used to combat fungal plant diseases. Consequently, excessive concentrations of fludioxonil may enter and accumulate over time in aquatic systems, harming (micro) organisms in several ways. Thus, it is of great importance to evaluate the potential toxic effects of fludioxonil using bioassays. In the present study, various in vitro assays were used to assess the possible effects of fludioxonil in human cells and aquatic microorganisms. For the investigation of the toxic effects of fludioxonil on freshwater microalgae, *Scenedesmus rubescens* and *Dunaliella tertiolecta* were exposed to various environmentally relevant concentrations of the fungicide for a period of 96 h. Fludioxonil at 50–200 μg L^−1^ significantly limited the growth of both microalgae, especially in the first 24 h of the exposure, where inhibitions up to 82.34% were calculated. The toxicity of fludioxonil was further evaluated via the Microtox test, and the studied fungicide was found to be less toxic for the bacteria *Aliivibrio fischeri*. Regarding human cells, the fludioxonil’s toxic and cyto-genotoxic effects were assessed using the Trypan blue exclusion test and the Cytokinesis Block MicroNucleus (CBMN) assay. Cell viability in all fludioxonil-treated concentrations was similar to control values according to the results of the Trypan blue exclusion test. However, the CBMN assay was used and revealed that fludioxonil had genotoxic potential in higher concentrations and exerted cytotoxic activity against human lymphocytes. Specifically, only the highest dose of fludioxonil, i.e., 10 μg mL^−1^, exerted genotoxic effects against human lymphocytes, whereas treatment with 0.5, 1, and 5 μg mL^−1^ did not lead to statistically significant induction of micronuclei (MN) frequencies compared with the control culture. However, fludioxonil-mediated cytotoxicity was statistically significant, which was demonstrated by the decreased CBPI (cytokinesis block proliferation index) values in all cases except for the lowest dose, i.e., 0.5 μg mL^−1^.

## 1. Introduction

Fludioxonil is a widely used phenylpyrrole fungicide in agricultural production that prevents diseases in vegetables, fruit trees, and crops [1,2,3]. It is frequently used in vineyards, including those in the Champagne region, to protect grapevines (*Vitis vinifera* L.) from fungal infections. It has been particularly effective against *Botrytis cinerea*, the agent responsible for grey mold. Fungal osmoregulation is upset by fludioxonil, resulting in cell death [4]. It is commonly co-applied with other fungicides, like triazoles, as part of integrated pest management techniques because of its broad-spectrum activity and effectiveness in preventing fungal resistance [5,6].

Derived from antibiotic pyrrolnitrin, fludioxonil is authorized for use in over 900 agricultural products [7,8]. Its widespread use and persistent nature have led to its presence in various environmental matrices and organisms [9,10,11]. In fact, high concentrations of the fungicide have been previously detected in agricultural soils and vineyard areas [12,13] as well as in indoor dust of farmworker households across Europe and Argentina in a recent study [14]. In addition to this, fludioxonil has been characterized as an endocrine disruptor for humans [15], while also adversely affecting various organisms [16]. Oxidative stresses through reactive oxygen species (ROS) production and mitochondrial dysfunction have already been proven as modes of action of fludioxonil [11,17]. WHO classifies it as moderately hazardous, while the European classification considers it toxic to aquatic life, with studies demonstrating its harmful effects on various organisms such as *Rhinella arenarum* and zebrafish (*Danio rerio*) [18,19].

Despite being mainly used in agriculture, the environmental fate of fludioxonil has drawn concern. After application, fungicide may pose a risk to organisms that are not intended targets by entering aquatic environments through surface runoff, leaching, and spray drift [20]. Its persistence in the environment and its detection in water bodies have been reported in earlier investigations [21,22]. The contamination of aquatic ecosystems by various agrochemicals has raised significant concerns regarding their impact on non-target organisms. Microalgae, being primary producers in aquatic environments, play a crucial role in ecological balance and are commonly used as bioindicators in ecological studies [23]. Their sensitivity to pollutants makes them ideal models for assessing the impact of environmental contaminants on photosynthesis, oxidative stress, and cellular metabolism.

Given the increasing presence of fludioxonil in various samples and its potential health implications, it is crucial to assess its cytotoxic and genotoxic effects on human cells [15,16]. Previous studies primarily focused on its impact on non-target aquatic organisms and microbial communities [20,21], with limited data available on its direct effects on human cells. Since human exposure can occur through dietary intake and occupational settings [19], a deeper understanding of its toxicological profile is necessary. Recent studies suggest that certain pesticides, including fludioxonil, may interfere with cellular homeostasis and contribute to adverse health effects [18]. However, comprehensive in vitro studies evaluating its cytotoxicity and genotoxicity in human cells remain scarce.

The Cytokinesis Block MicroNucleus (CBMN) assay is a simple, rapid, and robust assay used widely for the investigation of cyto-genotoxic potential of a plethora of compounds. Moreover, the sensitivity of human lymphocytes and their ease of culture following collection render them great candidates for the in vitro assessment of the toxic profiles of chemicals [23,24]. The CBMN assay detects micronuclei (MN) in the cytoplasm of once-divided interphase binucleated (BN) human lymphocytes, due to the inability of acentric chromosome fragments or whole chromosomes to migrate to the poles during the anaphase stage of the cell [25]. To evaluate the cytotoxic effect, the cell proliferation index (cytokinesis block proliferation index, CBPI) is calculated. Microalgae, such as *Scenedesmus rubescens* and *Dunaliella tertiolecta*, are well-documented bioindicators that are used widely to assess the effects of various pollutants due to various advantages that they possess, such as low cost and simplicity, among others [26,27]. In addition, the bioluminescence inhibition assay using the bacteria *Aliivibrio fischeri* is commonly used to evaluate the acute toxicity of chemical compounds. The bioluminescence produced by these bacteria is a metabolic activity that can be disrupted by toxicants, making it a reliable indicator of the chemicals [28].

This study investigates the cytotoxic and genotoxic effects of fludioxonil on human lymphocytes using the CBMN assay, alongside its impact on microalgae (*S. rubescens*, *D. tertiolecta*) and marine bacterium (*A. fischeri*). Given the increasing concerns regarding the environmental and health impacts of this widely used fungicide, in the present study, the potential effects on organisms of different trophic levels will be assessed, aiming to fill the knowledge gap regarding its potential risk to human cells and various aquatic microorganisms. By investigating its toxicological effects through multiple assays, this study not only provides essential data on its cytotoxic and genotoxic potential but also contributes to the broader understanding of pesticide safety. The novelty of this study lies in its comprehensive approach to evaluating fludioxonil’s toxicity across different biological models, providing a more holistic assessment of its environmental health effects.

## 2. Materials and Methods

### 2.1. Chemicals and Reagents

Trypan blue solution (0.4%), DPX mounting medium, Mitomycin C (MMC), and Giemsa were acquired by Sigma-Aldrich Chemical Co. (St. Louis, MO, USA), while Lymphosep (Lymphocyte separation media) was supplied by Biosera (Cholet, France). Ham’s F-10 medium, L-glutamine, Foetal bovine serum (FBS), and Phytohaemaglutinin (PHA) were purchased from Gibco (Paisley, Scotland) and Cytochalasin-B (Cyt-B) from Santa-Cruz (Heidelberg, Germany).

The microalgae species *S. rubescens* (strain SAG 5.95) was purchased from the bank SAG collection of the Gottingen University (Germany). *D. tertiolecta* (strain CCAP 19/6B) was purchased from Scottish Marine Institute, Oban, Argyll, Scotland.

BG-11 medium (Cyanobacteria BG-11 Freshwater Solution), F/2 medium (Guillard’s (F/2) Marine Water Enrichment Solution), sodium chloride, and ZnSO_4_·7H_2_O were purchased from Sigma-Aldrich (St. Louis, MO, USA). *A. fischeri* bacteria (Microtox^®^ Acute Reagent) and Microtox^®^ Reconstitution Solution were supplied by Modern Water (York, UK).

The rest of the chemicals and solvents were of the highest grade commercially available. The stocks of the compounds and solutions were kept at 4 °C, and the stock solution of Fludioxonil was kept at −20 °C.

### 2.2. Sample Preparation

Fludioxonil was in the form of powder. Thus, a stock of 1 mg mL^−1^ (1000 mg L^−1^) was prepared in ethanol, which is the organic solvent in which fludioxonil has the 2nd highest solubility (44 g mL^−1^). The stock solution was kept at −20 °C until use. The appropriate volumes of the stock solution were added to the cultures of human cells and microalgae in order the achieve the desired concentration. The final concentrations used for the cell viability assessment and the evaluation of cyto-genotoxic potential of fludioxonil were 0.5, 1, 5 μg mL^−1^ and 0.5, 1, 5, 10 μg mL^−1^, respectively. For both microalgae, the tested concentrations were 10, 50, 100, 200 μg L^−1^. For *A. fischeri,* a fludioxonil solution (100 mg L^−1^) was initially prepared from the stock solution and further tested in four dilutions (81.90, 40.95, 20.48, and 10.24 mg L^−1^).

### 2.3. Biological Testing

#### 2.3.1. Ethics Statement

The biological assays, using human peripheral blood lymphocytes, were based on international bioethics criteria. The study was approved by the Research Ethics Committee (REC) of the University of Patras (Ref. No. 7682/23 March 2022). Participants used as blood donors signed informed consent forms stating that they were not exposed to radiation, drug treatments, or any viral infection in the recent past.

#### 2.3.2. Whole Blood Collection

Whole blood samples were acquired from healthy non-smoking male donors (<30 years old) who were not exposed to radiation, drug treatment, or any viral infection in the recent past and were collected in heparinized tubes. All samples were treated appropriately to conduct the biological testing in all cases.

#### 2.3.3. Cell Viability

Lymphocytes were isolated from whole blood samples of each donor (N = 2), using Lymphosep, by a gradient centrifugation procedure [29], according to the manufacturer’s application protocol. Briefly, 3 mL of Lymphosep were added in sterilized centrifuge tubes (volume 15 mL), followed by 4 mL of whole blood samples primarily diluted in Ham’s F-10 medium (ratio 1:1). The centrifugation of the samples took place at room temperature for 15 min at 400 g, the lymphocyte layer was collected and subsequently diluted in equal volume of the culture medium.

Afterward, a washing step of the samples (200 g, 10 min, at room temperature ×2) was conducted, and the samples were resuspended in Ham’s F-10 medium for further analysis. Cells were treated for 3 h with different concentrations of fludioxonil (0.5, 1, 5 μg mL^−1^) at 37 °C in a humidified atmosphere of 5% CO_2_, and their viability was assessed using the Trypan blue exclusion test. Specifically, Trypan blue solution (0.4%) was used for cell staining, and cells were transferred to microscope slides and covered with cover slips. A light microscope (Carl Zeiss optical/light microscope; 100× magnification, Oberkochen, Germany) was used for slide analysis. A total of 1000 lymphocytes were counted in each case, and the number of viable (unstained) and non-viable (stained) cells was determined.

#### 2.3.4. CBMN Assay in Human Lymphocytes In Vitro

The cyto-genotoxic potential of fludioxonil at concentrations 0.5, 1, 5, 10 μg mL^−1^ in human lymphocytes was investigated by the in vitro Cytokinesis Block MicroNucleus (CBMN) assay using cytochalasin-B, according to standard procedures [25].

Scoring of micronuclei in binucleated cells was conducted automatically with the use of MNScore and MetaCyte system, based on the slide-scanning Metafer platform (MetaSystems, Altlussheim, Germany). At least 2000 binucleated (BN) cells with preserved cytoplasm were scored for each concentration tested. Moreover, cytotoxicity was determined via the calculation of the cytokinesis block proliferation index (CBPI) after counting 1000 cells for each experimental point, using the equation:

CBPI = [N1 + N2 + 3(N3 + N4)]/N,

where N1, N2, N3, and N4 represent the numbers of cells with one, two, three, and four nuclei, while N is the total number of cells [30].

### 2.4. Algal Biotest

Algal biotests were performed using the freshwater microalgae species *S. rubescens* (strain SAG 5.95), as well as the saltwater species *D. tertiolecta* (CCAP19/6B), which were cultivated in BG-11 medium (24 ± 1 °C, pH 8.3 ± 0.3) and F/2 without Si (24 ± 1 °C, pH 8.3 ± 0.3, salinity 35%), respectively, under constant illumination (4300 lux), according to the protocol 201 of OECD [31]. The algal cultures were exposed to different concentrations of the tested compound (10, 50, 100, 200 μg L^−1^) for a period of 96 h under conditions described in the protocol. The cell numbers using a Neubauer hemocytometer, the growth rate (μ), and the inhibition of growth rate (I %) were determined every 24 h. The results are the mean ± SD of two repetitions, in each case.

### 2.5. A. fischeri Bioluminescence Inhibition Test

The ecotoxicity of fludioxonil was also estimated using the *A. fischeri* Bioluminescence Inhibition Test. For this purpose, a Microtox Model 500 Toxicity Analyzer (Azur Environmental, Surrey, UK) was used, and fludioxonil solution (100 mg L^−1^) was tested in four dilutions in a medium containing 2% sodium chloride, and the bioluminescence was recorded after 5 and 15 min of exposure. An aqueous solution of 2% sodium NaCl and ZnSO_4_·7H_2_O was used as negative and positive controls, respectively.

### 2.6. Statistical Analysis

The final data are expressed as mean ± standard deviation (SD) of two independent experiments. Statistical analyses were performed using the SPSS 25 (2019) software package (Armonk, NY, USA: IBM Corp.). In the case of human lymphocyte cultures, a G-test for independence on 2 × 2 tables was used for MN data analysis, whereas the chi-square (*χ*^2^) test was used to analyze CBPI data. The significance of the differences between the variables obtained in the control and affected cells was assessed non-parametrically, using the Mann–Whitney U test (*p* < 0.05). Datasets were checked for homogeneity of variance (Levene’s test of equality of error variances). For the microalgae and *A. fischeri*, half maximal inhibitory concentrations (IC_50_) were calculated using probit analysis in SPSS based on the percentage inhibition values across concentrations.

## 3. Results

### 3.1. Toxic and Cyto-Genotoxic Effects of Fludioxonil in Human Lymphocytes

#### 3.1.1. Determination of Cell Viability in Fludioxonil-Treated Human Lymphocytes

According to the results of the Trypan blue exclusion test, the carrier solvent (ethanol) showed levels of cell viability similar to those that occurred in the control culture (Table 1). The same pattern was observed for human lymphocyte cultures treated with fludioxonil.

#### 3.1.2. Study of Cyto-Genotoxic Effects of Fludioxonil Using CBMN Assay

**Figure 1:**
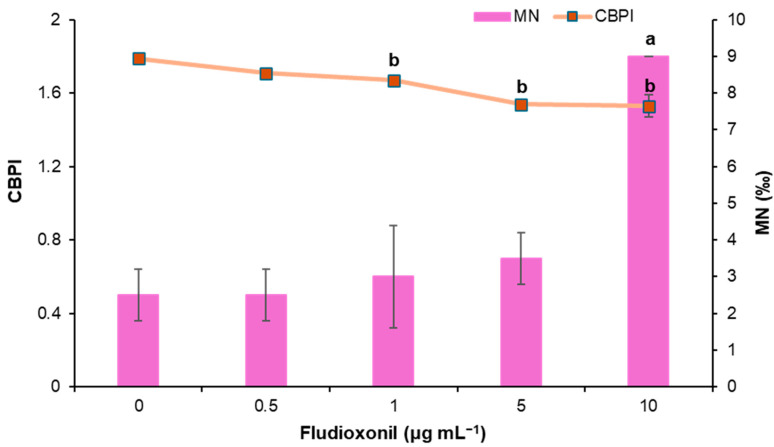
CBPI values and MN frequencies (‰) in human lymphocytes treated with 0.5, 1, 5 and 10 μg mL^−1^ of fludioxonil. Values with letters show significant difference in terms of CBPI and MN frequencies from the control culture (Mann–Whitney U-test).

Fludioxonil-treated cells were studied at four different concentrations (0.5, 1, 5, and 10 μg mL^−1^) to identify its potential risk of inducing genotoxic and cytotoxic effects in cultured human lymphocytes. Moreover, mitomycin-C was used as a positive control at a concentration of 0.05 μg mL^−1^. The results of the CBMN assay are depicted in Table 2 and Figure 1. According to the results, slightly increased MN frequencies were observed for the first three concentrations tested compared with the negative control, which were not statistically significant. Only the highest concentration of fludioxonil, i.e., 10 μg mL^−1^, was deemed genotoxic. MMC induced statistically significant MN induction as expected. Regarding the cytotoxic potential of fludioxonil, a dose-dependent decrease in CBPI and % cytostasis was observed in all concentrations compared with the control. All tested concentrations, except for the lowest (0.5 μg mL^−1^), exhibited statistically significant cytotoxic activity, with cytostasis ranging from 9.51 ± 0.72% to 32.57 ± 7.17% at the highest concentration.

### 3.2. Toxic Effects of Fludioxonil on S. rubescens and D. tertiolecta

**Figure 2:**
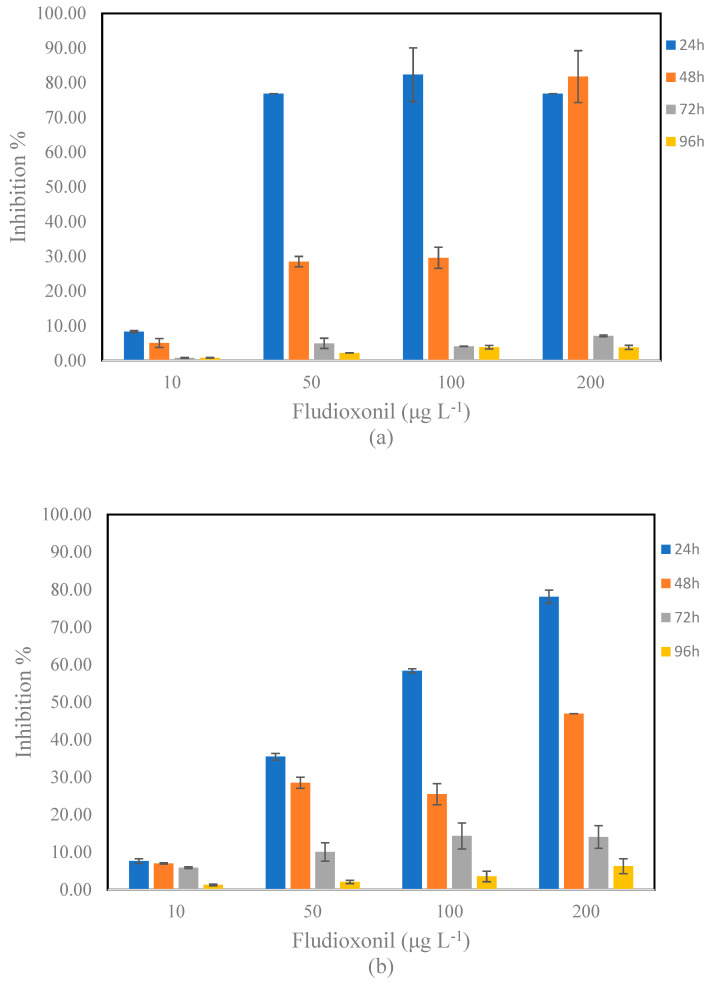
% Inhibition of growth rate of (**a**) *S. rubescens* and (**b**) *D. tertiolecta* after 24–96 h exposure to fludioxonil (10, 50, 100, and 200 μg L^−1^).

The effects of fludioxonil on *S. rubescens* and *D. tertiolecta* were evaluated at four different concentrations (10, 50, 100, and 200 μg L^−1^) and at four exposure times (24, 48, 72, and 96 h). The % Inhibition of the growth rate at each period of exposure is presented in Figure 2. For the calculation of the % Inhibition, the data presented in Figures S1–S8 were used.

At the lowest concentration (10 μg L^−1^), relatively low inhibitions for *S. rubescens* were observed at 24 and 48 h, which were decreased further at 72 and 96 h. At the concentration of 50 μg L^−1^, fludioxonil exhibited significant inhibition for *S. rubescens* at the early time points, with an inhibition of 76.88% at 24 h. However, this inhibition decreased markedly over time, with values dropping to 4.97% at 72 h and 2.22% at 96 h. The toxicity at 100 μg L^−1^ followed a similar trend, with inhibition percentages of 82.34% at 24 h, but again declining to 3.86% by 96 h. The highest concentration (200 μg L^−1^) displayed strong toxicity at 24 h and 48 h, with inhibition levels of 76.88% and 81.80%, but significantly decreased to 7.12 and 3.81% at 72 and 96 h, respectively. Regarding *D. tertiolecta*, a dose–response relationship of fludioxonil to microalgae was observed after 24 h of exposure. At the lowest concentration (10 μg L^−1^), relatively low inhibitions for *D. tertiolecta* were observed at all exposure times. In contrast, an inhibition of 78.20% was observed after exposure at the concentration of 200 μg L^−1^ at 24 h. *D. tertiolecta* exposed to 50, 100, and 200 μg L^−1^ of fludioxonil for 48 and 72 h showed significantly lower growth rates than those observed in 24 h. Table 3 compiles the IC_50_ values that indicate a shift in toxicity over time. For *S. rubescens*, at 24 and 48 h, the IC_50_ were 0.08 mg L^−1^, whereas at 72 and 96 h, the IC_50_ increased to 22.51 and 29.77 mg L^−1^, respectively. A quite similar trend was also observed for *D. tertiolecta.* The IC_50_ values remained low for 24 and 48 h (0.08 and 0.19 mg L^−1^), whereas they increased for 72 and 96 h (2.42 and 12.52 mg L^−1^).

### 3.3. Toxic Effects of Fludioxonil on A. fischeri

The toxicity of fludioxonil to *A. fischeri* was assessed at two different exposure times (5 and 15 min). The IC_50_ values calculated for *A. fischeri* are shown in Table 4. At the 5 min of exposure, the IC_50_ value for fludioxonil was 260.24 mg L^−1^, indicating that fludioxonil exhibits toxicity at this short exposure time but at high concentrations. However, at 15 min of exposure, all samples tested negative for inhibition, and therefore, the IC_50_ value could not be determined. This suggests that fludioxonil may not exhibit toxic effects on *A. fischeri* after a longer exposure time, or the toxicity may be below the levels at the concentrations tested.

## 4. Discussion

### 4.1. Toxic and Cyto-Genotoxic Effects of Fludioxonil on Human Lymphocytes

Fludioxonil is a widely used fungicide worldwide and constitutes an essential compound in many pesticide mixtures and agricultural products. Its presence has been confirmed in various environmental matrices, which will inevitably affect several organisms, including humans.

Despite its broad application, the scientific studies regarding its cyto-genotoxic and toxic effects on humans and other organisms are limited since most research focuses on its detection in the environment and the determination of its residues in agricultural products. In the present study, the toxic and cyto-genotoxic effects of fludioxonil were studied in human lymphocytes using the Trypan blue exclusion test and the Cytokinesis Block MicroNucleus (CBMN) assay. The concentrations tested were 0.5, 1, 5 μg mL^−1^ and 0.5, 1, 5, 10 μg mL^−1^ for the former and latter assay, respectively.

Cell viability in all fludioxonil-treated concentrations was similar to control values according to the results of the Trypan blue exclusion test. Only the highest concentration tested via the CBMN assay exerted genotoxic potential (10 μg mL^−1^), whereas a dose-dependent cytotoxic activity was induced, which was statistically significant in all concentrations except for 0.5 μg mL^−1^.

A few studies have investigated the genotoxic and/or mutagenic activity of fludioxonil. Graiet et al. [32] reported that fludioxonil caused DNA damage as demonstrated by the application of the comet assay on rat neural cells F98, which was dose-dependent and more pronounced at the highest dose, i.e., 10 μg mL^−1^. This aligns with the results of the present research, since fungicide exerted significant genotoxic potential at the same concentration against human lymphocytes. Oxidative stress induced by fludioxonil was suggested as a significant factor in the observed DNA damage. Graillot et al. [33] investigated the in vitro cytotoxic and genotoxic effects of pesticide mixtures present in the diet of the French population. Using the γ-H2AX assay and Comet assay, they investigated, among others, the detrimental effects of fludioxonil alone. Despite the absence of DNA damage in the comet assay, fludioxonil showed genotoxic activity in the γ-H2AX assay from approximately 1 μg mL^−1^, with more than 20% cytotoxicity on HepG2 cells from 2.5 μg mL^−1^. It was suggested that the genotoxicity of fludioxonil could be attributed to the formation of reactive intermediates during its biotransformation. Moreover, fludioxonil was found to be recombinogenic at 0.5 mg mL^−1^ using the somatic mutation and recombination test (SMART) in Drosophila wings [34]. Isidori et al. [35] used the Ames test to detect bacterial mutagenicity on *Salmonella typhimurium* TA98, TA100, and TA1535 and the SOS chromotest to detect the primary DNA damage on *Escherichia coli* PQ37, regarding the genotoxic activity of various pesticides, including fludioxonil. It was found to be positive for both *S. typhimurium* and *E. coli*, indicating mutagenic potential.

Regarding the toxic and cytotoxic potential of fludioxonil, several researchers have investigated its effects and its modes of action. Human lymphocytes, specifically Jurkat T cells and Ramos B cells, were used to investigate the toxic profile of fludioxonil [36]. A decrease in cell viability was observed at the highest concentration of Jurkat T cells (10^−5^ M) at 24 h and in a dose-dependent manner at 48 h (10^−8^–10^−5^ M). A similar pattern was observed for Ramos B cells only at 48 h, with a dose-dependent decrease in cell viability at concentrations of 10^−7^–10^−5^ M. Human lung fibroblasts (WI-26 cells) treated with fludioxonil did not present any changes in their viability. Furthermore, cell cycle arrest was induced in both T and B cells. Consequently, it was shown that fludioxonil induced cytotoxicity in human immune cells via apoptosis by inhibiting cell proliferation and the cell cycle arrest. The cytotoxic activity of fludioxonil against human immune cells is in accordance with our results. The viability and cell proliferation of human immortalized embryonic kidney (HEK293) cells were examined against fludioxonil via the 3 (4.5-dimethylthiazol-2-yl)-2.5-diphenyltetrazolium bromide (MTT) and Trypan blue exclusion assays. According to the results, the proliferation of the cells was not significantly affected, but cell death was induced, albeit at concentrations significantly higher than the ones used in our study, i.e., approximately 0.5–44 mg mL^−1^ [34]. Fludioxonil exhibited cytotoxic activity on human neuroblastoma (SH-SY5Y) cells and glial cell line (U251) in vitro, leading to a significant reduction in cellular ATP, affecting mitochondrial membrane potential and causing oxidative stress and apoptosis. The effects were more pronounced in SH-SY5Y cells. In general, the lipophilic nature of fludioxonil could facilitate the penetration of the human blood barrier to reach the central nervous system [37]. A dose-dependent decrease in rat neural cells F98 viability was observed, indicating the cytotoxic potential of the fungicide, with an IC_50_ of approximately 10 μg mL^−1^ [32]. Furthermore, a significant increase in ROS was observed, as well as lipid peroxidation. In addition to this, treatment with fludioxonil led to cell cycle arrest and disruption of the cytoskeleton structure. The generation of free radicals may play a significant role in the induction of apoptosis, which could be induced via the mitochondrial pathway. Additionally, recent research reported fludioxonil as a potential anti-androgenic compound capable of inducing developmental toxicity, specifically shortening of anogenital distance in rodent models, thus expanding the concern beyond cytotoxicity to possible endocrine-disrupting effects [38].

It is noteworthy to mention that the cyto-genotoxic and toxic potentials of fludioxonil vary depending on the organism/cell used in each study and the assays applied, as well as the concentrations tested. However, it is generally reported that fludioxonil increases oxidative stress, causes mitochondrial dysfunction, and induces apoptosis. Further research should be conducted to elucidate the detrimental effects of fludioxonil both in vitro and in vivo, as well as its long-term impact on different organisms.

### 4.2. Toxic Effects of Fludioxonil on S. rubescens and D. tertiolecta

According to the results, fludioxonil has a high initial toxicity, and its effects lessen over time. The observed acute ecotoxicities toward algae can be correlated with the enhanced production of ROS, damaging microalgae cells’ structure and functionality. Fludioxonil was studied regarding its toxic effects against the microalgae *Chlorella vulgaris*, and its mode of action was investigated [22]. The half maximal effective concentration (EC_50_) of fludioxonil at 96 h was 1.87 mg L^−1^. The photosynthetic pigment content was reduced, and a significant increase in ROS levels was recorded, which led to the induction of oxidative stress. Morphological changes were also observed in the cell structure, and apoptosis occurred. Wang et al. [18] investigated among others, the toxic effects of fludioxonil on zebrafish (*Danio rerio*). Biochemical, molecular, and genetic analyses in different stages of zebrafish development indicate an increase in ROS levels and an induction of apoptosis, which demonstrates the toxic potential of the fungicide in the specific organism. The toxicity of fludioxonil was also investigated on non-target aquatic plants, duckweed, *Lemna minor,* and on algae *Scenedesmus acutus*. The IC_50_ values of fludioxonil for *L. minor* and *S. acutus* were >100 and 4.85 mg L^−1^, respectively. Thus, the tolerance against fludioxonil and its potential toxicity varies depending on the species affected [39]. Moreover, fludioxonil has demonstrated significant chronic toxicity toward sediment-dwelling macroinvertebrates in long-term exposure studies, further highlighting its broader ecotoxicological impact on aquatic ecosystems [40].

According to the United Nations classification of ecotoxicity [41], compounds are categorized based on their EC_50_ (or IC_50_) values as follows:

Highly toxic: EC_50_ (IC_50_) ≤ 1 mg L^−1^.

Toxic: 1 mg L^−1^ < EC_50_ (IC_50_) ≤ 10 mg L^−1^.

Harmful to aquatic organisms: 10 mg L^−1^< EC_50_ (IC_50_) ≤ 100 mg L^−1^.

Based on these IC_50_ values, fludioxonil would be classified as highly toxic at 24 h and 48 h for both tested microalgae. At 72 h, fludioxonil is characterized as harmful and toxic for *S. rubescens* and *D. tertiolecta*, respectively. Moreover, it is characterized as harmful to both tested microalgae at 96 h. The decrease in the toxicity that is observed with the increase in the exposure time can be correlated with the potential transformation of fludioxonil in aqueous media and the production of transformation products, which, however, retain aquatic toxicity [42]. Possible adaptive responses of the algae may also contribute to this effect [26].

### 4.3. Toxic Effects of Fludioxonil on A. fischeri

The Microtox^®^ assay was performed using the marine bioluminescent bacteria *A. fischeri*, and the bioluminescence inhibition was assessed after 5 and 15 min of incubation. Based on the results, fludioxonil generated low inhibition effects on *A. fischeri*. Consequently, fludioxonil cannot be characterized as toxic or harmful to the tested bacteria. Available ecotoxicological data in the literature support that there is a relationship between toxicity and the physicochemical properties of pesticides [43]. In the study conducted by Verdisson et al. [39], the low toxicity of fludioxonil was reported to be correlated with the poor solubility of the compound. Furthermore, the relatively higher resistance of *A. fischeri* may be attributed to fundamental differences in cell structure, metabolic pathways, and ecological niches between prokaryotic and eukaryotic organisms. *A. fischeri* is a heterotrophic marine bacterium, and many xenobiotics, including fungicides such as fludioxonil, often target eukaryotic specific processes such as photosynthesis, oxidative stress responses, and cell division that are absent in bacterial systems [44].

Several studies support the observation that microalgae are typically more sensitive to chemical pollutants than marine bacteria. Lomba et al. [44] reported that *Raphidocelis subcapitata* exhibited significantly higher sensitivity than *A. fischeri* when exposed to six different pharmaceuticals, despite both showing concentration-dependent responses. Similarly, Rodríguez Pérez et al. [45] found that pesticide-contaminated water from the Alqueva reservoir exhibited high toxicity toward algae but no measurable inhibition of bioluminescence in *A. fischeri*, emphasizing the greater resilience of bacterial bioluminescence systems to certain pollutants. In another study, Jesus et al. [46] demonstrated that both algae and daphnids displayed acute responses to contaminants at much lower concentrations than *A. fischeri*, which may relate to differences in cellular uptake, membrane permeability, and molecular targets affected by toxicants. Thus, the lower sensitivity of *A. fischeri* observed in this study aligns with existing literature and highlights the importance of incorporating diverse test species from multiple trophic levels in ecotoxicological risk assessments of pesticides such as fludioxonil.

## 5. Conclusions

In the present study, a combination of toxicity bioassays in aquatic micro-organisms along with the application of in vitro toxicity assays in human lymphocytes was used to assess the potential effects of fludioxonil in the environment and humans. According to the results, fludioxonil demonstrated both genotoxic and cytotoxic potential under the experimental conditions of this study. The analysis of CBPI showed that fludioxonil induces a statistically significant cytotoxic effect at all tested concentrations except for the lowest one, compared with the negative control. Both tested marine microalgae species (*S. rubescens* and *D. tertiolecta*) were significantly affected after exposure to fludioxonil, at 24 and 48 h. Although the toxicity was decreased at prolonged exposure periods (72 and 96 h), fludioxonil can be classified as harmful and toxic for *S. rubescens* and *D. tertiolecta* at 72 h, respectively, whereas it is characterized as harmful for both tested microalgae at 96 h. In contrast, fludioxonil provoked low inhibition effects on *A. fischeri*. Consequently, its potential toxicity varies depending on the species affected and highlights the need for ecotoxicity studies that use different bioindicators.

## Figures and Tables

**Table 1:** Evaluation of human lymphocyte viability after treatment with different concentrations of fludioxonil. The results expressed as % viability are mean ± SDs from 2 independent experiments in each case.

**Fludioxonil (μg mL^−1^)**	**% Viability**
0	99.75 ± 0.07
0.5	99.45 ± 0.07
1	99.50 ± 0.14
5	98.45 ± 0.07
**Solvent**	**% Viability**
0 (Ethanol)	99.80 ± 0.00

**Table 2:** Frequencies of micronucleated binucleated cells (BNMN), micronuclei (MN), cytokinesis block proliferation index (CBPI) values, and % cytostasis in cultured human lymphocytes which have been treated with fludioxonil and mitomycin-C (MMC) (0.05 μg mL^−1^). The results are expressed as mean ± SDs from 2 independent experiments in each case.

Treatment (μg mL^−1^)	BNMN	MN	*p*	CBPI	*p*	Cytostasis (%)
0	2.50 ± 0.70	2.50 ± 0.70	-	1.79 ± 0.00	**-**	**-**
0.5	2.50 ± 0.70	2.50 ± 0.70	-	1.71 ± 0.01	0.053	9.51 ± 0.72
1	3.00 ± 1.40	3.00 ± 1.40	1.00	1.67 ± 0.02 ^b^	8.47 × 10^−5^	14.83 ± 2.87
5	3.50 ± 0.70	3.50 ± 0.70	0.550	1.54 ± 0.01 ^b^	1.66 × 10^−14^	31.05 ± 0.72
10	9.00 ± 0.00	9.00 ± 0.00 ^a^	0.001	1.53 ± 0.06 ^b^	8.07 × 10^−19^	32.57 ± 7.17
MMC (0.05)	53.50 ± 6.40	54.00 ± 5.70 ^b^	2.7 × 10^−52^	1.65 ± 0.00 ^b^	5.42 × 10^−7^	18.00 ± 0.90

BNMN: micronucleated binucleated cells; MN: micronuclei; *p*: statistical significance. CBPI: cytokinesis block proliferation index; MMC: Mitomycin-C. ^a^ Significant difference compared with control at *p* < 0.01. ^b^ Significant difference compared with control at *p* < 0.001.

**Table 3:** IC_50_ values (mg L^−1^, 95% CI) of fludioxonil for *S. rubescens* and *D. tertiolecta*.

Exposure Time (h)	*S. rubescens*	*D. tertiolecta*
24	0.08 (0.07–0.1)	0.08 (0.06–0.09)
48	0.08 (0.07–0.09)	0.19 (0.15–0.27)
72	22.51 (1.65–2.53 × 10^17^)	2.42 (0.90–25.51)
96	29.77 (2.57–5.28 × 10^15^)	12.52 (2.11–23,067.66)

**Table 4:** IC_50_ values (mg L^−1^, 95% CI) of fludioxonil for *A. fischeri*.

Exposure Time (min)	IC_50_ (mg L^−1^)
5	260.24 (152.06–1026.50)
15	n.d.

## Data Availability

Data are contained within the article and Supplementary Materials.

## Supplementary Materials

The following supporting information can be downloaded at https://www.mdpi.com/article/10.3390/toxics13050358/s1, Figure S1. Fludioxonil effects on (a) cell density (×10^4^ cells mL^−1^) and (b) algal growth rate (μ, day^−1^) of *S. rubescens* after 24 h of exposure; Figure S2. Fludioxonil effects on (a) cell density (×10^4^ cells mL^−1^) and (b) algal growth rate (μ, day^−1^) of *S. rubescens* after 48 h of exposure; Figure S3. Fludioxonil effects on (a) cell density (×10^4^ cells mL^−1^) and (b) algal growth rate (μ, day^−1^) of *S. rubescens* after 72 h of exposure; Figure S4. Fludioxonil effects on (a) cell density (×10^4^ cells mL^−1^) and (b) algal growth rate (μ, day^−1^) of *S. rubescens* after 92 h of exposure; Figure S5. Fludioxonil effects on (a) cell density (×10^4^ cells mL^−1^) and (b) algal growth rate (μ, day^−1^) of *D. tertiolecta* after 24 h of exposure; Figure S6. Fludioxonil effects on (a) cell density (×10^4^ cells mL^−1^) and (b) algal growth rate (μ, day^−1^) of *D. tertiolecta* after 48 h of exposure; Figure S7. Fludioxonil effects on (a) cell density (×10^4^ cells mL^−1^) and (b) algal growth rate (μ, day^−1^) of *D. tertiolecta* after 72 h of exposure; Figure S8. Fludioxonil effects on (a) cell density (×10^4^ cells mL^−1^) and (b) algal growth rate (μ, day^−1^) of *D. tertiolecta* after 96 h of exposure.

## Abbreviations

The following abbreviations are used in this manuscript:

SD	Standard deviation
CI	Confidence interval
CBMN	Cytokinesis Block MicroNucleus
MN	Micronuclei
CBPI	Cytokinesis block proliferation index
WHO	World Health Organization
BN	Binucleated
BNMN	Micronucleated binucleated cells
MMC	Mitomycin-C
SMART	Somatic mutation and recombination test
ROS	Reactive oxygen species
