# Supplementary material for: Assessment of Potential Toxic Effects of Fungicide Fludioxonil on Human Cells and Aquatic Microorganisms
Source: Toxics. 2025 Apr 30;13(5):358. doi: 10.3390/toxics13050358 (PMC12116062; doi:10.3390/toxics13050358)
Supplement: Supplementary file 1 [file toxics-13-00358-s001.zip › toxics-3532287-supplementary.pdf]

toxics

Supplementary material

Assessment of potential toxic effects of fungicide fludioxonil on human cells and aquatic microorganisms

Maria Antonopoulou[1]*, Anna Tzamaria[1], Sotiris Papas[2], Ioanna Efthimiou[2], Dimitris Vlastos[2]

[1]Department of Sustainable Agriculture, University of Patras, Seferi 2, GR-30131, Agrinio, Greece; mantonop@upatras.gr (M.A.); annatz408@gmail.com (A.T.)

[2]Department of Biology, Section of Genetics, Cell Biology and Development, University of Patras, GR-26504, Patras, Greece; sotirispapas@outlook.com (S.P.), iefthimiou@upatras.gr (I.E.); dvlastos@upatras.gr (D.V.)

*Correspondence: mantonop@upatras.gr; Tel.: (+30) 26410-74114

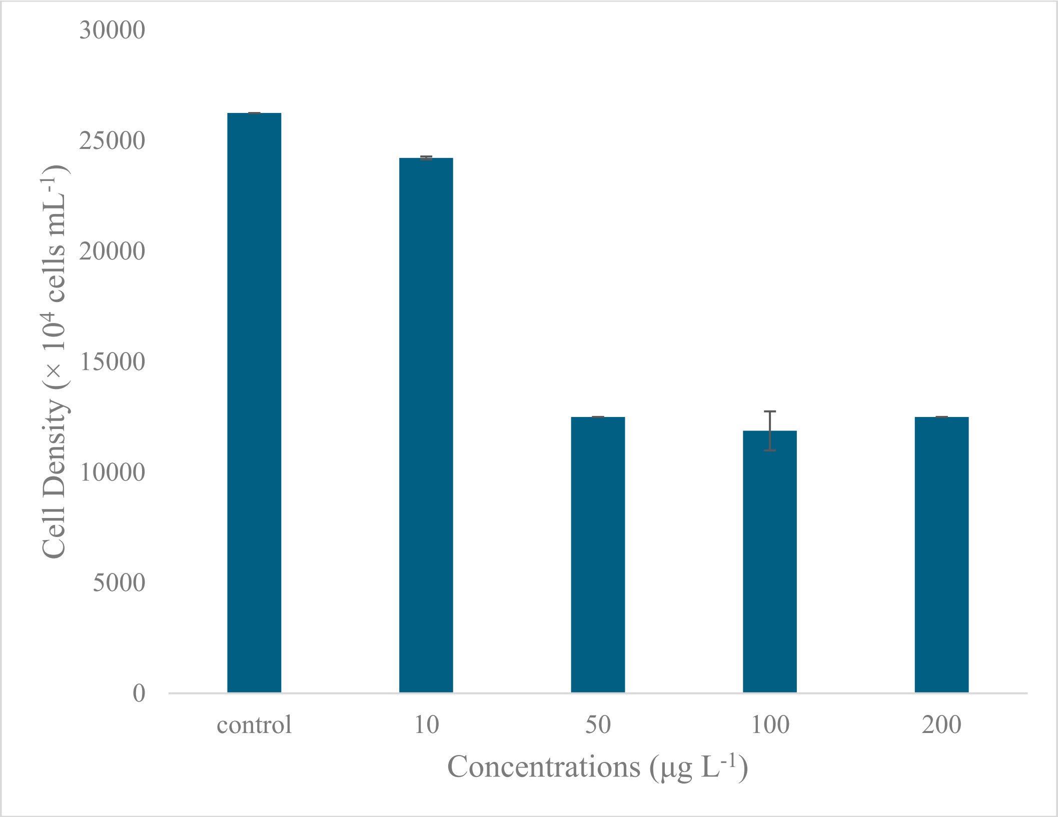

(a)

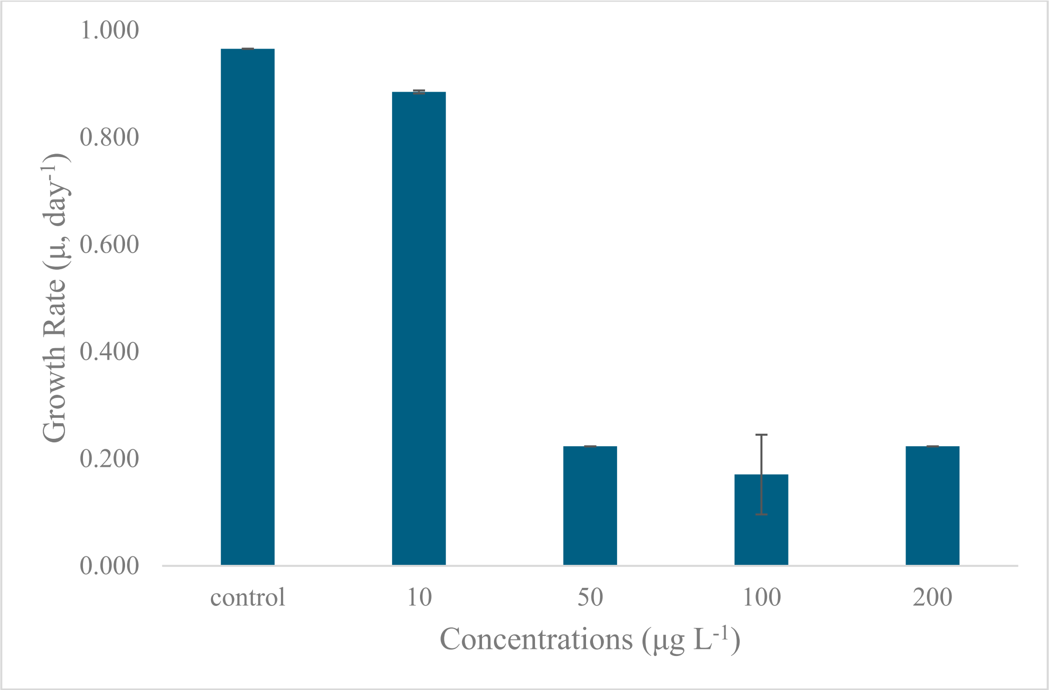

(b)

Figure S1. Fludioxonil effects on (a) cell density (× 10^4 cells mL^{-1}) and (b) algal growth rate (µ, day^{-1}) of *S. rubescens* after 24 h of exposure.

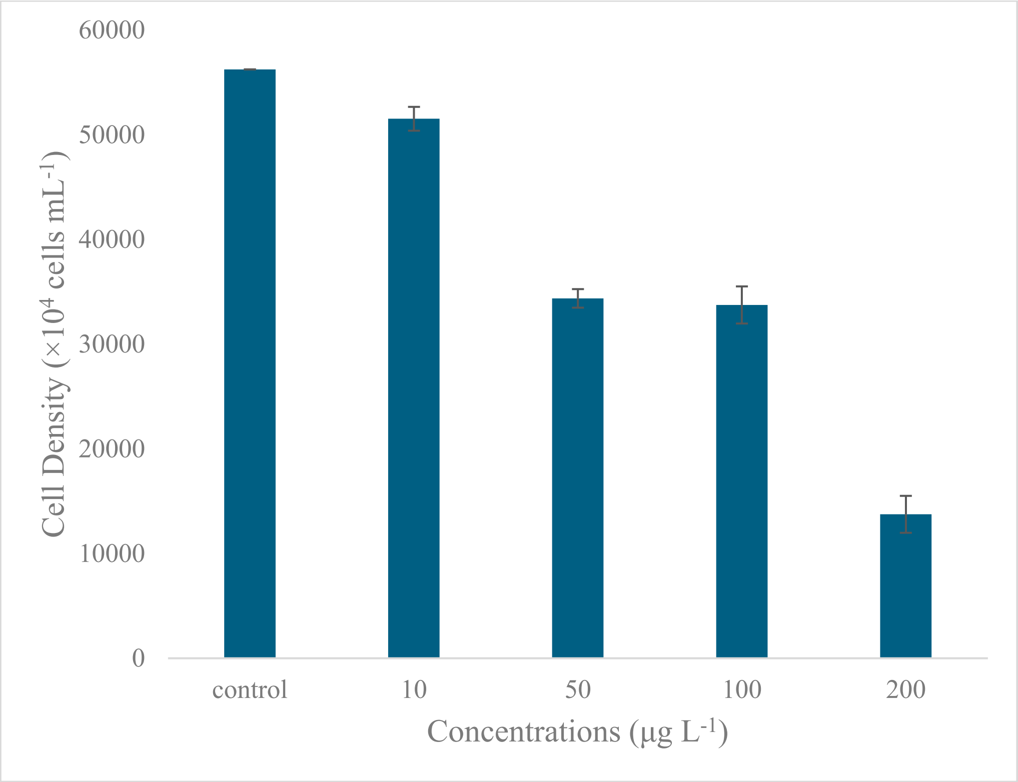

(a)

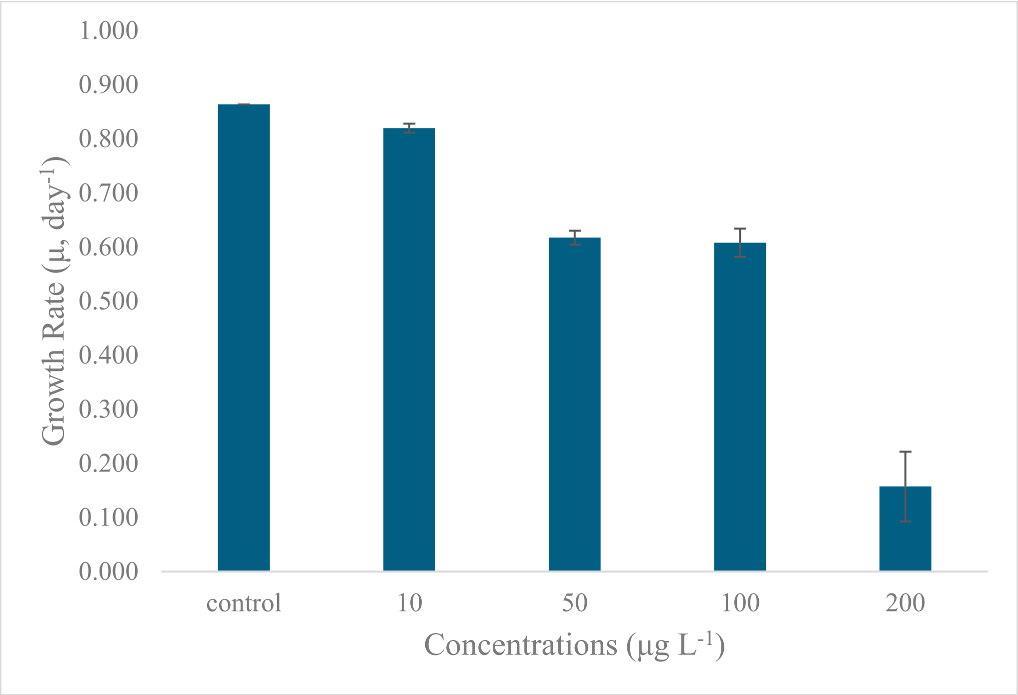

(b)

Figure S2. Fludioxonil effects on (a) cell density (× 10^4 cells mL^{-1}) and (b) algal growth rate (μ, day^{-1}) of *S. rubescens* after 48 h of exposure.

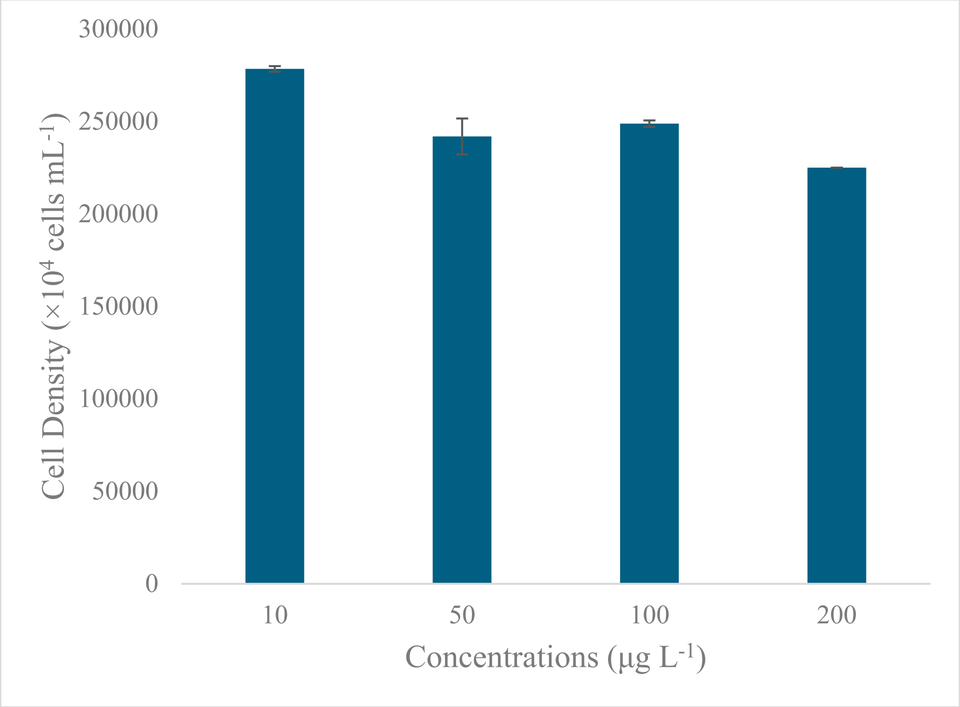

(a)

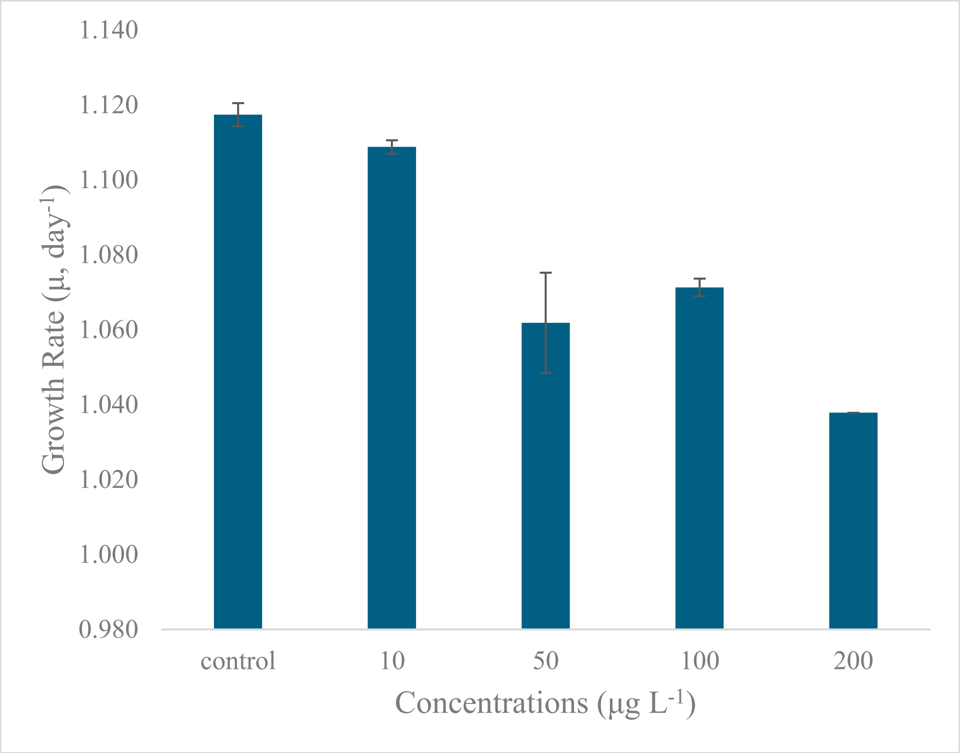

(b)

Figure S3. Fludioxonil effects on (a) cell density (× 10^4 cells mL^{-1}) and (b) algal growth rate (μ, day^{-1}) of *S. rubescens* after 72 h of exposure.

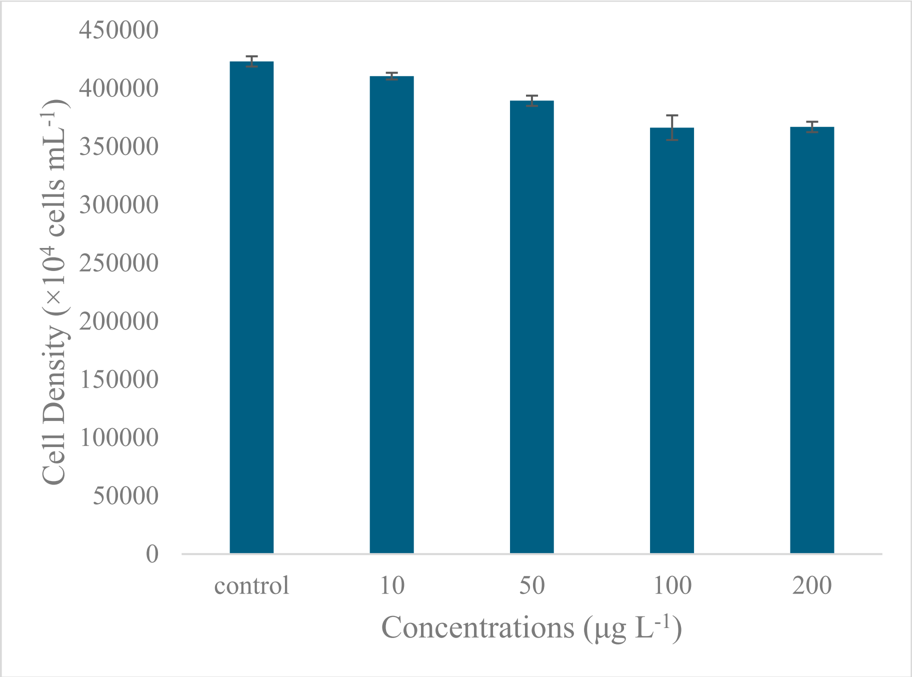

(a)

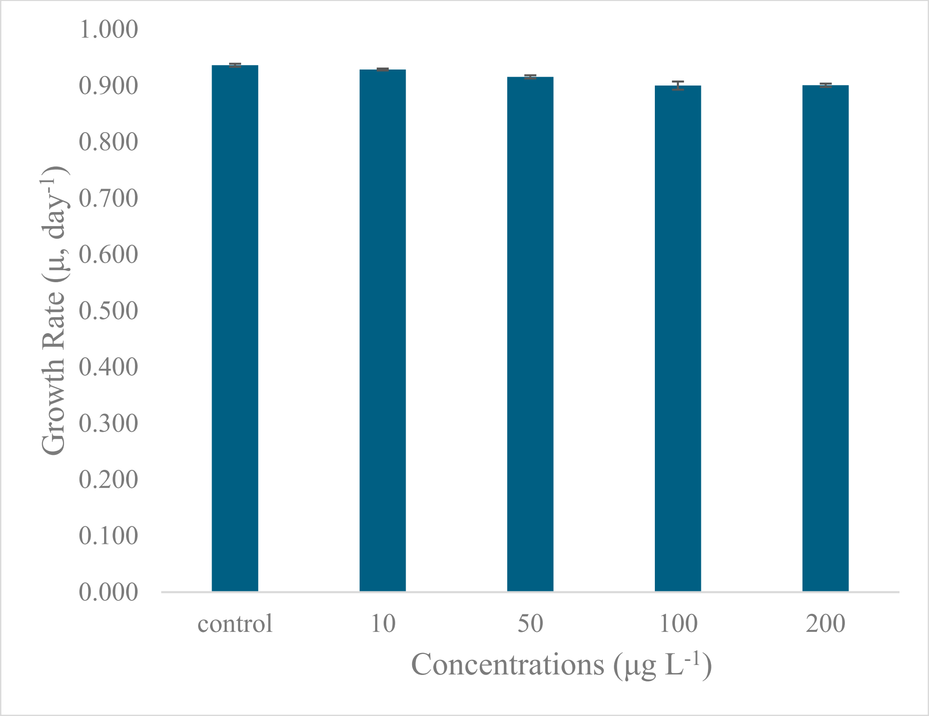

(b)

Figure S4. Fludioxonil effects on (a) cell density (× 10^4 cells mL^{-1}) and (b) algal growth rate (µ, day^{-1}) of *S. rubescens* after 96 h of exposure.

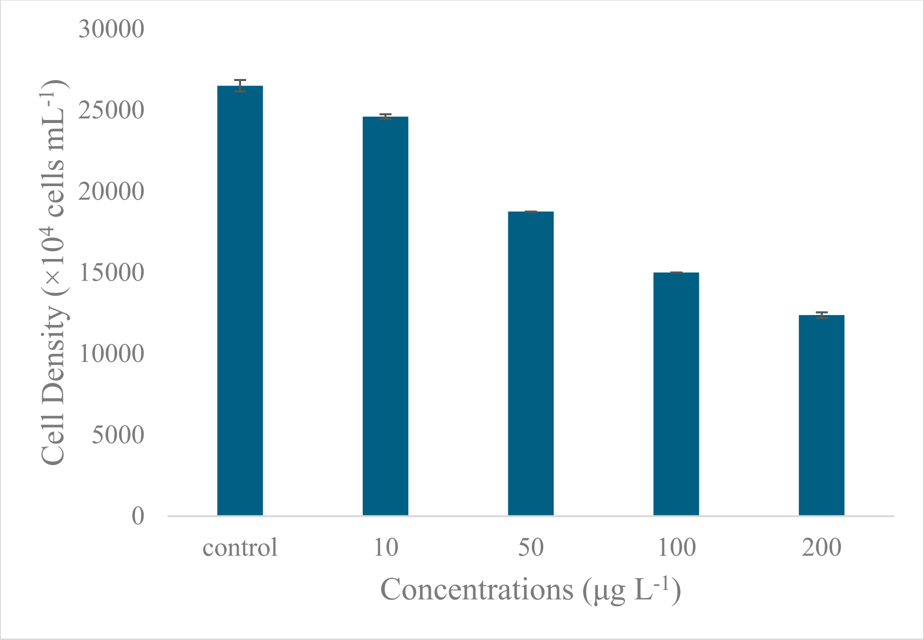

(a)

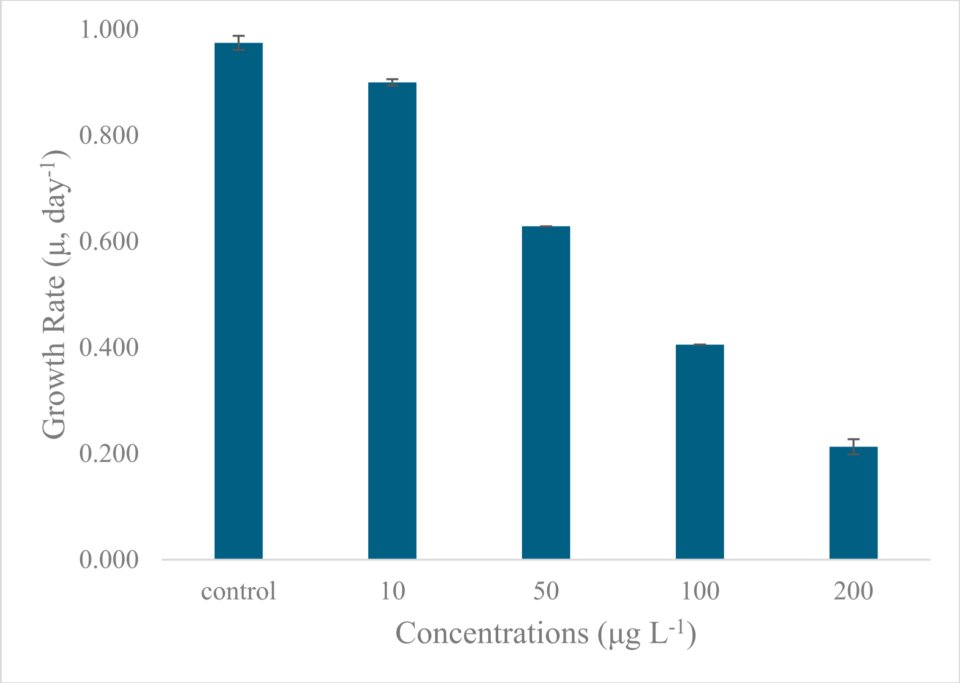

(b)

Figure S5. Fludioxonil effects on (a) cell density (× 10^4 cells mL^{-1}) and (b) algal growth rate (µ, day^{-1}) of *D. tertiolecta* after 24 h of exposure.

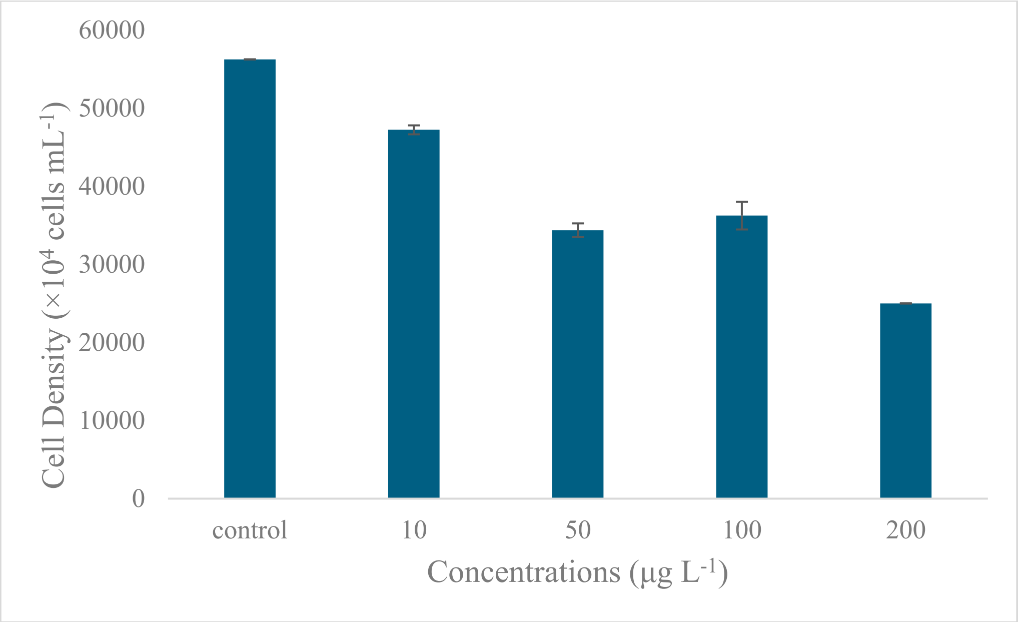

(a)

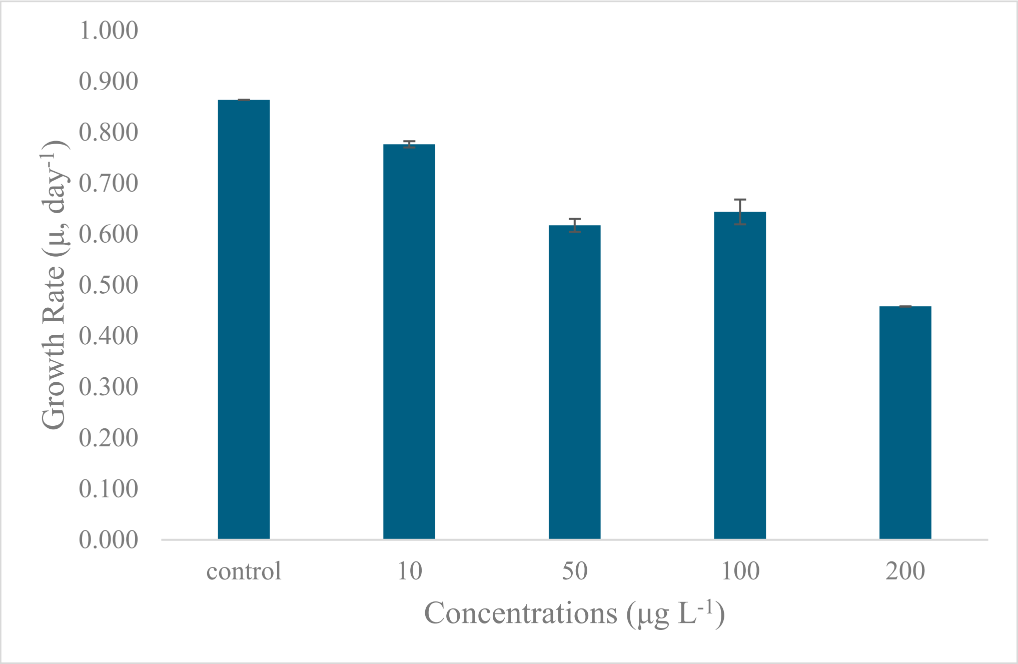

(b)

Figure S6. Fludioxonil effects on (a) cell density (× 10^4 cells mL^{-1}) and (b) algal growth rate (µ, day^{-1}) of *D. tertiolecta* after 48 h of exposure.

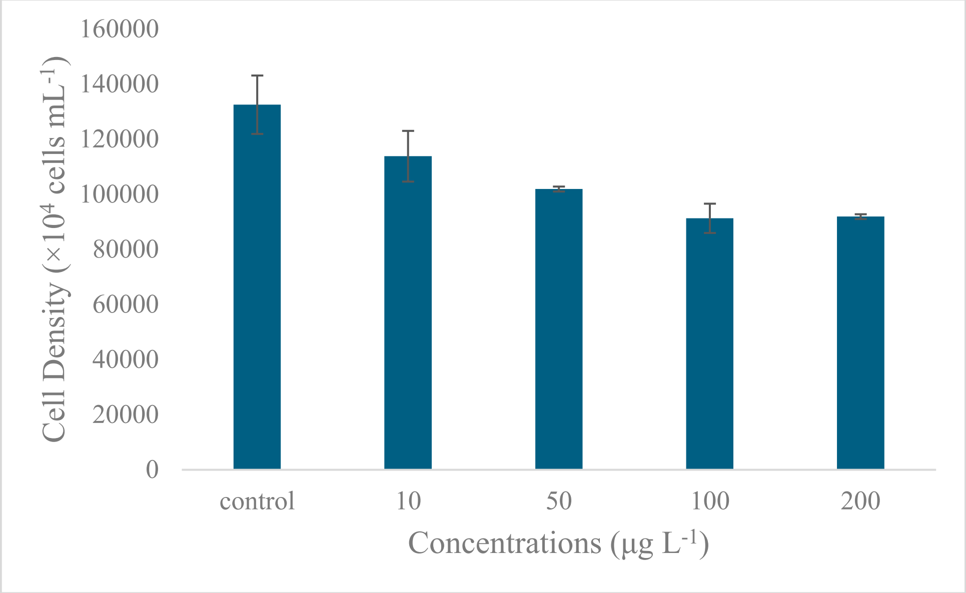

(a)

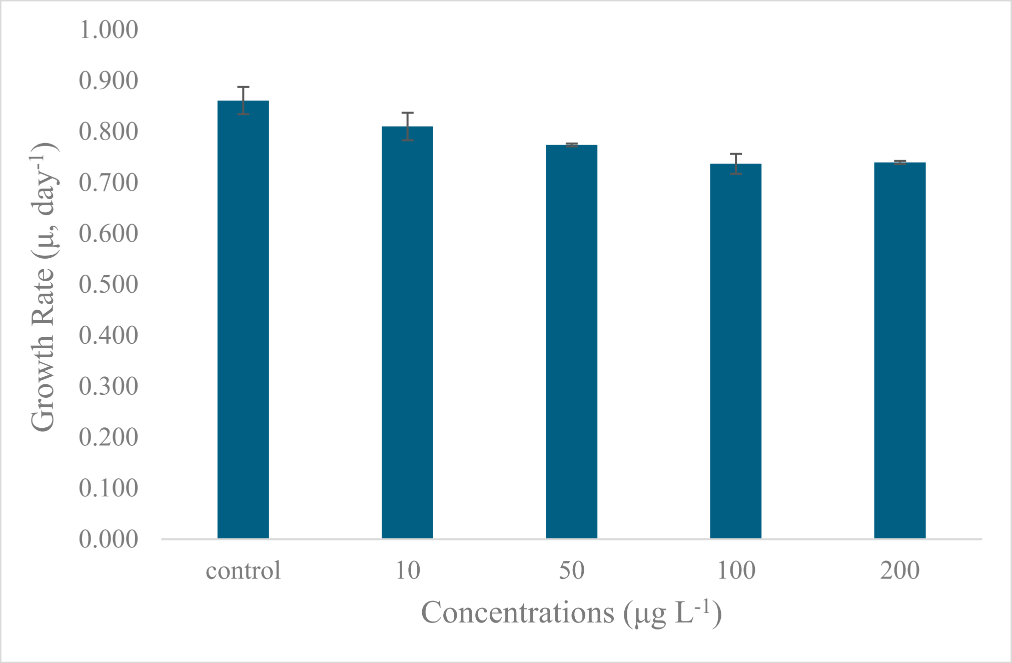

(b)

Figure S7. Fludioxonil effects on (a) cell density (× 10^4 cells mL^{-1}) and (b) algal growth rate (µ, day^{-1}) of *D. tertiolecta* after 72 h of exposure.

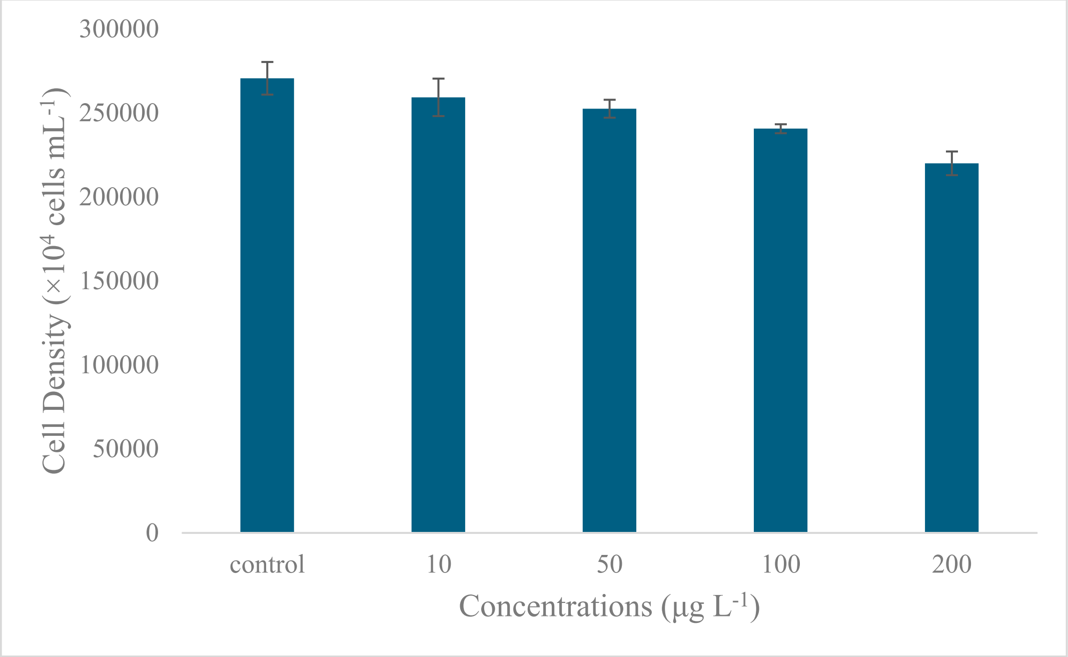

(a)

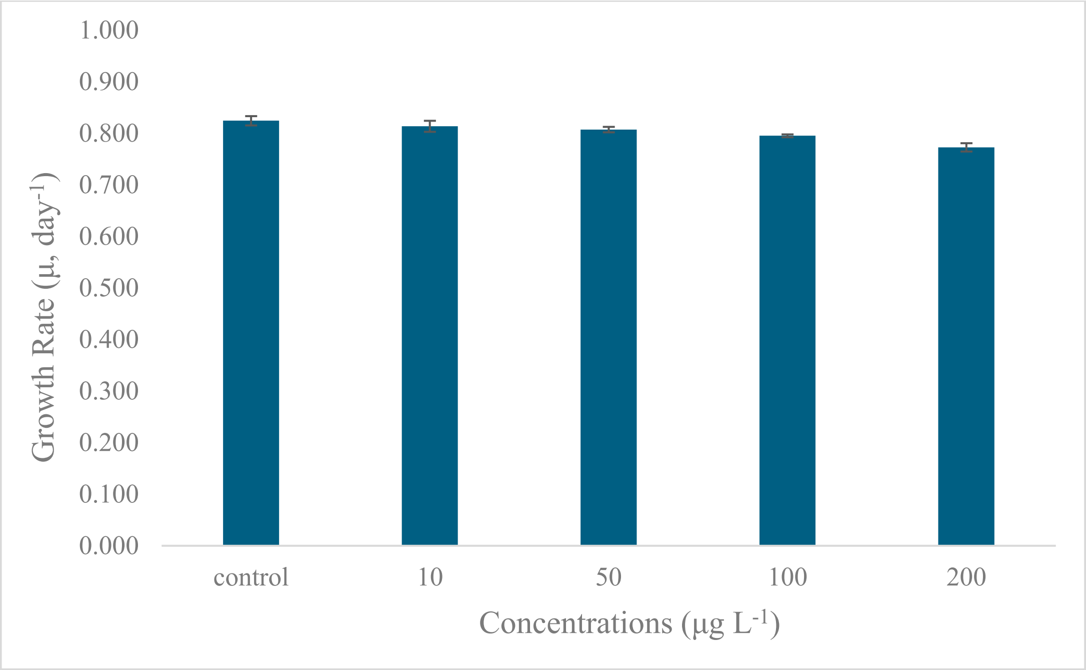

(b)

Figure S8. Fludioxonil effects on (a) cell density (× 10^4 cells mL^{-1}) and (b) algal growth rate (µ, day^{-1}) of *D. tertiolecta* after 96 h of exposure.
